# Food Insecurity and Nutritional Challenges in Adolescent and Young Adult Cancer Survivors in the U.S.A.: A Narrative Review and Call to Action

**DOI:** 10.3390/nu15071731

**Published:** 2023-04-01

**Authors:** Callie Ogland-Hand, Timothy H. Ciesielski, Katherine Daunov, Melanie K. Bean, Nora L. Nock

**Affiliations:** 1 Mary Ann Swetland Center for Environmental Health, Case Western Reserve University, Cleveland, OH 44106, USA; 2 Department of Population and Quantitative Health Sciences, Case Western Reserve University, Cleveland, OH 44106, USA; 3 Oncofertility and Young Adult Oncology, University Hospitals Seidman Cancer Center, Cleveland, OH 44106, USA; 4 Children’s Hospital of Richmond at Virginia Commonwealth University, Richmond, VA 23219, USA; 5 Population and Cancer Prevention Program, Case Comprehensive Cancer Center, Cleveland, OH 44106, USA

**Keywords:** adolescent and young adult (AYA) cancer survivors, food insecurity, nutrition security, malnutrition, financial insecurity

## Abstract

Advancements in cancer treatments over the past several decades have led to improved cancer survival in adolescents and young adults (AYAs, ages 15–39 years). However, AYA cancer survivors are at an increased risk for “late effects”, including cardiovascular, pulmonary and bone diseases as well as fatigue, infertility and secondary cancers. The treatments for cancer may also alter taste, lead to nutritional deficiencies and increase financial burdens that, when taken together, may increase the risk of food and nutrition security in AYA cancer survivors. Furthermore, although AYAs are often merged together in cancer survivorship studies, adolescents and young adults have distinct developmental, psychosocial and pathophysiological differences that may modify their risk of nutritional challenges. In this narrative review and “Call to Action”, rationale is provided for why there is a need to better understand nutritional challenges and food insecurity in AYA cancer survivors as a special population. Then, recommendations for next steps to advance knowledge and policy in this field are provided. In particular, integrating screening for food and nutrition insecurity and enhancing awareness of existing resources (e.g., the Supplemental Nutrition Assistance Program, SNAP) might help AYA cancer survivors combat nutritional deficiencies and reduce late effects while improving their overall survival and quality of life.

## 1. Adolescent and Young Adult (AYA) Cancer Survivors “Late Effects”

Over 89,000 new cancer cases are diagnosed each year in adolescents and young adults (AYAs, ages 15–39 years) [1]. Advancements in cancer treatments over the past several decades have decreased overall cancer mortality and increased cancer survival rates with five-year survival rates for all cancers combined, ranging from 83–86% in AYAs [1]. However, AYA cancer survivors are at an increased risk of developing longer term adverse health effects (commonly referred to as “late effects”), including cardiovascular, pulmonary, metabolic and bone diseases and secondary cancers as well as cognitive impairments, which can reduce their overall survival and quality of life [2,3,4,5]. More specifically, several studies have shown that AYA cancer survivors are at a higher risk of developing cardiomyopathy, stroke and premature ovarian failure compared to AYAs without cancer [6]. Treatments for cancer may also induce taste and smell disturbances, which can, in turn, alter eating behaviors and lead to nutritional deficiencies, including malnutrition and/or obesity in AYA cancer survivors [7,8,9,10]. Many AYA cancer survivors also experience fatigue, which can persist for years after treatment [1,4]. Therefore, it is not surprising that many AYA cancer survivors do not meet the recommended physical activity and dietary guidelines [11,12,13], further increasing their risk of secondary cancers and other chronic diseases.

Despite a higher risk for late effects, an estimated 50–60% of AYA survivors report not having received any information from their clinical team regarding their increased risk of late effects [14]. In addition, compared to older adult patients (ages 40 years and older) with cancer, AYAs have a higher risk of long-term and late effects, including cardiovascular disease, infertility, sexual dysfunction and secondary cancers [15,16]. AYAs are also more likely than older patients with cancer to experience delays in diagnosis for secondary cancers because of higher rates of medical uninsurance and lower rates of screening [17,18].

AYA cancer survivors may experience higher rates of psychosocial and mental health issues. In particular, AYA cancer survivors have more poor mental health days compared to healthy controls [19] and many have anxiety, depression and post-traumatic stress disorders, which can exacerbate fears of recurrence [20,21]. Both cancer diagnoses and treatments can present AYAs with challenges interacting with peers and developing socially [20]. Many AYA cancer survivors report experiencing loneliness [22] and some have described difficulties trusting that they can find social support when needed [20]. Further, many AYA cancer survivors have medical insurance-related distress [20,23].

## 2. Nutritional Challenges in AYA Cancer Survivors

In addition to the complex medical and psychosocial challenges described above, AYA cancer survivors are vulnerable to nutritional deficiencies and undernourishment as a consequence of cancer treatments [10]. A recent study reported that 8% of AYA cancer patients were underweight (BMI < 18.5 kg/m^2^) before treatment, a number that rose to 20% during treatment [10]. Malnourishment during cancer treatment is associated with decreased chemotherapy tolerance, increased infection rates and poorer overall survival [10]. There is a high prevalence of vitamin D deficiency among patients with cancer of any age and, in particular, in AYAs diagnosed with acute lymphocytic leukemia (ALL) and testicular cancer [24,25,26]. Nutritional deficiencies can continue into survivorship. Many young survivors experience deficiencies in vitamin D, calcium, folate and iron [27]. Vitamin D and other nutritional insufficiencies can have long-lasting, negative implications on bone health and, although the evidence is less clear, these deficiencies may also increase the risk of adverse metabolic and pregnancy outcomes [28,29,30]. Calcium deficiency may compound the problems with osteoporosis and folate and iron deficiencies and may increase the risk of adverse maternal and infant outcomes [31,32,33,34].

It is also important to recognize that malnutrition can coexist in people with obesity [35]. Indeed, obesity may mask the presence of malnutrition in AYA cancer survivors with obesity. Obesity rates ranging from 21–40% have been reported, particularly among AYA survivors of ALL cancers [36], and these rates are higher than those observed among their healthy counterparts [37,38,39]. Although specific data and the underlying mechanisms in AYA cancer survivor obesity are not known, self-reported taste and smell alterations are prevalent in 17–93% of cancer patients [7,40]. Furthermore, some adult cancer survivors with obesity have enhanced neurophysiological responses to consuming high-calorie, nutrient weak foods [41], which may be similar in AYA cancer survivors with obesity. However, to our knowledge, no prior studies have examined the neurobehavioral response to food cues in AYA cancer survivors, highlighting an area in need of future research.

## 3. Food Insecurity in AYA Cancer Survivors

Given the increased risk of nutritional challenges and food insecurity, there is an urgent need to better estimate the prevalence of food insecurity in this growing population of over 633,000 AYA cancer survivors in the U.S. [1,6]. In 1996, the United Nations Food and Agriculture Organization (FOA) published the following definition of food security: “Food security is a situation that exists when all people, at all times, have physical, social and economic access to sufficient, safe, and nutritious food that meets their dietary needs and food preferences for an active and healthy life” [42]. Food insecurity is contemporarily defined as a lack of consistent food volume and quality [43].

In the U.S., 20% of all families with children are food insecure [43]. However, only a few studies have estimated food insecurity in adult cancer survivors (18 years and older), and these studies estimate that food insecurity ranges from 4% to 26% among all adult cancer survivors [44,45]. Another study, using NHANES data, found that the odds of food insecurity decreased with increasing age, such that cancer survivors in their 50s and older were significantly less likely to be food insecure than those in their 20s and 30s [46]. Another study in adult cancer survivors of any age found that those who were food insecure compared to those who were food secure were more likely to forgo or delay their cancer treatment adherence/follow-up medical appointments [47]. Interestingly, a recent qualitative study interviewing 41 registered dieticians (RDs) working with cancer survivors across the U.S. found that although they have significant concerns regarding food insecurity in their cancer patients, most RDs expressed that they do not use a validated tool to identify food insecurity and most were not aware that any tools for measuring food insecurity exist [48].

To our knowledge, no prior studies have evaluated food insecurity specifically in AYA cancer survivors, who may be among the most vulnerable to the effects from food insecurity. Furthermore, it is important to recognize that nutrition insecurity can exist even in the presence of food security [49,50]. Formally, nutrition security is defined as “having consistent access, availability, and affordability of foods and beverages that promote well-being and prevent (and if needed, treat) disease” [51]. Thus, studies are needed to better understand food insecurity as well as *nutrition security* in AYA cancer survivors who are already at a higher risk of nutritional deficiencies and chronic diseases from their cancer treatments.

## 4. Financial Insecurity and Mental Health Concerns in AYA Cancer Survivors

Approximately two-thirds of AYA survivors report that cancer has had a negative financial impact on them [52]. The costs of cancer treatments are increasing [53], and economic hardships from cancer may have lasting effects that can persist for years [54]. Compared with same-age counterparts, AYA survivors have excess medical expenses of over USD 3000 annually and excess productivity losses of over USD 2000 annually [55]. Furthermore, the burden of these financial hardships is greater among AYA cancer survivors compared to older cancer survivors [55,56,57].

**Figure 1:**
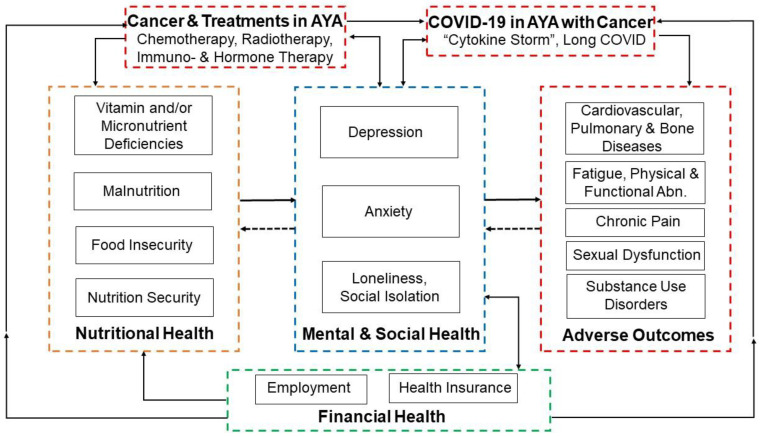
**Food and Nutrition Insecurity in Adolescent and Young Adult (AYA) Cancer Survivors:** Treatments for cancer may exacerbate nutritional deficiencies and adverse health outcomes (“late effects”) in AYA cancer survivors. Higher levels of financial insecurity and mental health issues common in this population as well as the COVID-19 pandemic may exacerbate effects of food and nutrition insecurity.

Cancer-related financial burdens are associated with increased risk of depression, anxiety and lower health-related quality of life [57], which may further modify the putative adverse effects of food and nutrition insecurity on disease (Figure 1). In particular, Anestin et al. (2018) found that depression was prevalent in about 20% of AYA patients [58], and Ander et al. (2016) reported a 29% prevalence of anxiety symptoms in AYA cancer survivors who were 10 years post-chemotherapy [59].

## 5. Potential Exacerbation of Food Insecurity by COVID-19 in AYA Cancer Survivors

The COVID-19 pandemic has exacerbated the complications of cancer treatments and survivorship, particularly among AYAs, as illustrated in Figure 1. Although studies specific to AYA cancer patients are lacking, the “cytokine storm” from COVID-19 can cause high morbidity and mortality in patients with cancer, particularly those who are treated with immunotherapies [60]. There is also emerging evidence that chronic vitamin D deficiency, which is common in AYA cancer survivors [27], may increase the immune and inflammatory dysfunctions that lead to poor COVID-19 outcomes [61,62]. The relationship between nutrient deficiencies and food insecurity is complicated by income status [51,63,64,65] and how these interactions impact COVID-19 infection among cancer patients is still being uncovered. Interestingly, hesitancy for COVID-19 vaccination persists in AYA survivors despite their increased chances of severe COVID-19 outcomes [66,67]. The pandemic additionally disrupted and delayed clinical care and exacerbated psychosocial and financial challenges, including financial distress, loneliness and other mental health issues in AYA cancer survivors [68,69,70].

## 6. Differences between Adolescent and Young Adult Cancer Survivors

Typically, adolescents (15–18 years of age) and young adults (19–39 years of age) are grouped together as “AYA cancer survivors”; however, they have certain distinct pathophysiological and developmental differences. For example, the type of cancer that they encounter varies substantially by age. Adolescents have a higher proportion of common “childhood cancers”, including Hodgkins lymphoma and ALL, and young adults have a higher proportion of common “adult cancers”, including thyroid, testicular, breast, uterine and colorectal cancers [1]. Rates of survival also vary by age. Leukemia continues to be the leading cause of cancer death in AYAs aged 20 to 29 years, while for those aged 30 to 39 years, colorectal and uterine corpus cancers represent the leading causes of death [1]. Developmentally, adolescents often struggle with identity, figuring out how to “fit in” while dealing with hormonal changes, self-consciousness and increases in risk-taking behavior [71]. However, young adults are focused on building and maintaining intimate relationships, developing careers and finding stable housing [71]. Adolescents may be more likely to live with a caregiver/parent, whereas young adults are more likely to be living on their own. Thus, their risk of food insecurity and nutritional challenges may be different and, hence, there is a need to consider adolescents and young adult cancer survivors as separate subgroups.

## 7. Recommendations for Future Research and Policy to Address Food and Nutrition Security in AYA Cancer Survivors

Our narrative review highlights that there is currently a dearth of information on food insecurity specifically among AYA cancer survivors. This is concerning because they have unique challenges as a result of developmental and pathophysiological differences. These challenges can be exacerbated by financial hardships from cancer treatments, survivorship care as well as COVID-19 related pandemic issues, ultimately compromising their ability to access and consume healthy, nutrient dense foods. We believe steps should be taken to better support the nutritional needs and overall health and wellbeing of the more than 633,000 AYA cancer survivors living in the U.S. [1,6]. In particular, future studies should estimate the prevalence of food insecurity in AYA cancer survivors specifically and should consider young adults (15–18 years of age) and adolescents (19–39 years of age) as distinct sub-populations.

Screening for food insecurity is a “missing piece” in the current management of cancer survivors [72]. Thus, we recommend that widespread screening for food insecurity and *nutrition security* be initiated using existing efficient tools, such as the Food Insecurity Vital Sign [73] and Malnutrition Screening Tool [74], and/or new questionnaires modified to meet the unique needs of AYA cancer survivors. Furthermore, these screening tools could be administered when cancer patients are routinely screened for other measures, such as distress, or as part of an AYA Needs Assessment Service Bridge (AYA NA-SB) [75]. In addition, a working group with representation from national organizations, such as the National Comprehensive Cancer Network (NCCN), Oncology Nursing Society, American Society of Clinical Oncologists and Oncology Nutrition Dietetic Practice Group, could be formed to help set guidelines for food insecurity screening for AYA as well as other oncology patient subgroups.

In addition, we recommend that campaigns and educational support programs be instituted to increase AYA cancer survivors’ awareness of existing food support programs. For example, AYA cancer survivors may be unaware of general food assistance programs, such as Supplemental Nutrition Assistance Program (SNAP) [76] and the Special Supplemental Nutrition Program for Women, Infants and Children (WIC) [77]. Perhaps, AYA survivorship navigators in the hospital/clinic setting could help patients with SNAP or WIC service enrollment and/or advising patients of other local food pantry and meal service programs. Furthermore, formal referral services to nutrition professionals (e.g., RDs) should be in place to address food and nutrition insecurities when identified during screening.

Clearly, a multi-disciplinary approach will be needed to integrate screening for food and nutrition security and provide the necessary corresponding follow-up programs to address food and nutrition insecurities in AYA cancer survivors. Below, we outline our list of recommended next steps for future research and policy initiatives:

1. Conduct studies to estimate food and nutrition security in AYA cancer survivors to better understand risks in adolescents and young adults as distinct subgroups.
2. Initiate campaigns and educational support programs that specifically target AYA cancer survivors to enhance awareness of existing food support programs (e.g., SNAP and WIC).
3. Conduct routine screening for food insecurity, nutrition security and malnutrition in AYA cancer survivors as part of survivorship plans or, preferably, starting at cancer diagnosis and continuing throughout survivorship during clinical follow-up visits.
4. Provide professional nutritional support services to AYA cancer survivors, with a particular focus on those who are identified to be at increased risk during screening.
5. Provide special provisions in SNAP tailored to AYA cancer survivors with a focus on the needs of young adult cancer survivors to maximize benefits (e.g., similar to the provisions for the elderly).
6. Initiate campaigns to reduce the stigma associated with food insecurity screening and the use of food support services.

## 8. Conclusions

Our narrative review highlights that there is currently a dearth of information on food insecurity specifically among AYA cancer survivors. This is concerning because they have unique challenges as a result of developmental and pathophysiological differences. These challenges can be exacerbated by financial hardships from cancer treatments and survivorship care as well as COVID-19-related pandemic issues, ultimately compromising their ability to access and consume healthy, nutrient dense foods. Improvements to the infrastructure and tools needed to screen for food and nutrition insecurity need to be developed. In the interim, campaigns could be immediately initiated to help increase awareness of existing food support programs, which may assist in lowering the risk of malnutrition and other chronic diseases while improving overall health and quality of life in AYA cancer survivors.

## Data Availability

Not applicable.
